# Identification of a Novel Protein-Protein Interaction Motif Mediating Interaction of GPCR-Associated Sorting Proteins with G Protein-Coupled Receptors

**DOI:** 10.1371/journal.pone.0056336

**Published:** 2013-02-18

**Authors:** Olivier Bornert, Thor C. Møller, Julien Boeuf, Marie-Pierre Candusso, Renaud Wagner, Karen L. Martinez, Frederic Simonin

**Affiliations:** 1 Institut de Recherche de l'ESBS, Biotechnology and Cellular Signaling, Universite de Strasbourg-CNRS UMR 7242 / laboratory of excellence MEDALIS, Illkirch, France; 2 BioXtal, Saint Félix, France; 3 Bio-Nanotechnology and Nanomedicine Laboratory, Department of Chemistry and Nano-Science Center, University of Copenhagen, Copenhagen, Denmark; University of São Paulo, Brazil

## Abstract

GPCR desensitization and down-regulation are considered key molecular events underlying the development of tolerance *in vivo*. Among the many regulatory proteins that are involved in these complex processes, GASP-1 have been shown to participate to the sorting of several receptors toward the degradation pathway. This protein belongs to the recently identified GPCR-associated sorting proteins (GASPs) family that comprises ten members for which structural and functional details are poorly documented. We present here a detailed structure–function relationship analysis of the molecular interaction between GASPs and a panel of GPCRs. In a first step, GST-pull down experiments revealed that all the tested GASPs display significant interactions with a wide range of GPCRs. Importantly, the different GASP members exhibiting the strongest interaction properties were also characterized by the presence of a small, highly conserved and repeated “GASP motif” of 15 amino acids. We further showed using GST-pull down, surface plasmon resonance and co-immunoprecipitation experiments that the central domain of GASP-1, which contains 22 GASP motifs, is essential for the interaction with GPCRs. We then used site directed mutagenesis and competition experiments with synthetic peptides to demonstrate that the GASP motif, and particularly its highly conserved core sequence SWFW, is critically involved in the interaction with GPCRs. Overall, our data show that several members of the GASP family interact with GPCRs and highlight the presence within GASPs of a novel protein-protein interaction motif that might represent a new target to investigate the involvement of GASPs in the modulation of the activity of GPCRs.

## Introduction

G protein-coupled receptors (GPCRs) represent one of the most diversified protein families in humans. They modulate a large panel of physiological processes making them unrivalled targets for development of new therapeutic agents. GPCRs translate extracellular stimuli into intracellular signals, and the intensity and duration of these are determined by complex regulation mechanisms. Internalization, whereby agonist-activated GPCRs are rapidly silenced by translocation from the cell surface to endocytic compartments, represents a central event for the modulation of receptor activity [1]. Upon internalization, GPCRs can be recycled back to the membrane or degraded. Although mechanisms that govern the postendocytic fate of GPCRs are not fully understood, several proteins have been shown to modulate this phenomenon via a direct interaction with their carboxyl-terminal intracellular tail (C-tail) [2]–[5]. One of these regulatory proteins is GPCR-associated sorting protein 1 (GASP-1), which was identified in a yeast two-hybrid screen using the δ-opioid receptor (DOR) cytoplasmic C-tail as bait [6], [7] and has been shown to be involved in the sorting of receptors that are quickly degraded following agonist-promoted internalization [7]. This phenomenon has been proposed to form the molecular basis for analgesic tolerance to cannabinoids [8], [9]. GASP-1 was also found to interact with numerous other receptors both *in vitro* [6], [7], [10] and *in vivo* [11], [12]. Although, its has been proposed that the binding epitope for GASP-1 to these receptors is large an covers major parts of the cytoplasmic C-tail of receptors (Heydorn et al., 2004), we have shown that helix 8, located near the seventh transmembrane helix, is critically involved in this interaction [6]. Concerning GASP-1, little is known about which region within this protein is required for its interaction with GPCRs.

GASP-1 is part of a novel protein family of ten members that were identified by sequence homology searches [6], [13]. The carboxyl-terminal 250 amino acids (AA) region of GASP-1 displays remarkable sequence identity with the nine other members of the family. Furthermore, GASP genes, except GASP-8, contain a single coding-exon and are located within two 200-kilobase clusters on two adjacent contigs on chromosome X, suggesting that they arose from a common ancestral gene [13], [14]. Altogether, these data indicate that GASPs do indeed form a protein family and might display similar functions. However, except GASP-1 and GASP-2, interaction of the other members of the family with GPCRs has not been investigated so far.

In this study, we present a detailed structure–function relationship analysis of the molecular interaction between GASPs and GPCRs. GST-pull down experiments revealed that, besides GASP-1 and GASP-2, different members of the GASP family can interact with a wide range of GPCRs. Using surface plasmon resonance (SPR) analysis with two full-length GPCRs in solution and co-immunoprecipitation of GPCRs expressed in HEK293 cells, we further showed that the central domain of GASP-1 is critical for its interaction with GPCRs. Finally, we identified within this domain and in several other GASPs a conserved and repeated sequence of 15 amino acids that we called “GASP motif” and demonstrated that this motif plays a critical role in the interaction with GPCRs, both by site directed mutagenesis and competition experiments with synthetic peptides. Overall, our results demonstrate that GASPs indeed represent a novel family of GPCR-interacting proteins that can be divided into two subfamilies depending on the presence of the GASP motif. Our data clearly show that this sequence represents a novel protein–protein interaction motif that is critical for the interaction between GASPs and GPCRs.

## Materials and Methods

### Materials

The pGEX-2T and pGEX-4T3 prokaryotic expression vectors were purchased from Amersham Biosciences (GE Healthcare) and the pcDNA3.1 eukaryotic expression vector was from Invitrogen. The cDNA encoding GASP-3 was obtained from the Kazusa DNA Research Institute (Chiba, Japan; http://www.kazusa.or.jp/huge/) and cDNAs encoding GASP-6, -7, and -9 cloned into pBluescript II SK (+) were from Invitrogen. Radiolabelled [^35^S]-Methionine was purchased from ICN. GASP peptide (RVKQEPRFEEEVIIGSWFWAEKEA), control peptide (RVKQEPRFEEEVIIGAAAAAEKEA) and scramble peptide (AVEWIQEVFWEKRKPEEFGIERAS) were from Genecust with a ≥85% purity grade.

### Production of GST-fused Proteins

cDNAs encoding the cytoplasmic C-tail of 14 GPCRs and 2 one-transmembrane receptors were cloned into the pGEX-2T prokaryotic expression vector downstream the GST sequence. The following pGEX constructs were engineered: pGEX-DOR encoding residues 314–372 of the δ-opioid receptor (referring to Swiss-Prot accession number U10504), pGEX-MOR encoding residues 334–400 of the µ-opioid receptor (L29301), pGEX-KOR encoding residues 326–380 of the κ–opioid receptor (U17298), pGEX-ORL_1_ encoding residues 315–370 of the opioid receptor-like 1 (X77130), pGEX-M_1_ encoding residues 414–460 of the muscarinic M_1_ acetylcholine receptor (X52068), pGEX-M_2_ encoding residues 436–466 of the muscarinic M_2_ acetylcholine receptor (M16404), pGEX- ADRB1 encoding residues 373–477 of the β_1_ adrenergic receptor (J03019), pGEX-ADRB2 encoding residues 322–413 of the β_2_ adrenergic receptor (P07550), pGEX-CALCR encoding residues 407–490 of the calcitonin receptor (L00587), pGEX-CNR2 encoding residues 295–360 of the cannabinoid type 2 receptor (P34972), pGEX-5HT_7_ encoding residues 380–445 of the 5-hydroxytryptamine 7 receptor (L21195), pGEX-H_2_ encoding residues 284–349 of the histamine 2 receptor (S57565), pGEX-TXA_2_ encoding residues 304–343 of the α isoform of the thromboxane A_2_ receptor (D38081), pGEX-FZ_4_ encoding residues 493–537 of the frizzled 4 receptor (AB032417), pGEX-TGF_β_ encoding residues 805–849 of the type III transforming growth factor b receptor (L07594), pGEX-IGF_1_ encoding residues 1268–1367 of the insulin growth factor I receptor (X04434). Free GST was produced by using the pGEX-4T3 expression vector without insert. The same plasmid was used to clone the cDNA encoding a central domain of GASP-1, corresponding to amino acids 380 to 1073, downstream the GST sequence. All these plasmids were transformed into the *Escherichia coli* BL21 strain and expression was induced using 1****mM of isopropylthiogalactoside for 2 h at 37°C. Bacteria were then pelleted, resuspended in PBS supplemented with cOmplete protease inhibitors (Roche), lysed at 1.5 kbar using a basic Z cell disrupter (constant system) and finally the lysates were cleared by centrifugation (10,000g, 15 min, 4°C).

### [^35^S]-labelled GASPs in vitro Production

cDNAs encoding GASP-1 and GASP-2 were cloned into the pcDNA3 eukaryotic expression vectors [6]. cDNAs encoding GASP-3, -6, -7, and -9 were subcloned into pcDNA3.1 vectors. All the clones were controlled by DNA sequencing. The *in vitro* production was performed by using the TNT Quick-coupled Transcription/Translation T7 kit (Promega) in presence of [^35^S]-Methionine according to the instructions from the manufacturer.

### GST-pull Down Assay

GST-fusion proteins were immobilized on glutathione-coupled Sepharose beads (GE Healthcare) for 1 h at 4°C and incubated with [^35^S]-labelled *in vitro* translated GASP proteins or their truncated or mutated forms in ice-cold binding buffer containing: 20 mM Tris-HCl pH 7.4, 150 mM NaCl, 1 mM EDTA, 1 mM DTT, 10% glycerol and 1% Triton X-100. The mixture was incubated for 1 h at 4°C with gentle rocking. The beads were washed five times with the same ice-cold buffer, resuspended in 30 µl SDS-PAGE sample buffer, incubated 10 min at 65°C, and pelleted for 60 s at 3000 g. The supernatants were then analyzed by SDS-PAGE, the gels were stained with Coomassie blue to visualize GST-fusion proteins, dried and analyzed using a Phosphor-imager (Personal Molecular Imager FX, Biorad) to visualize [^35^S]-labelled GASPs. Quantification was performed with the Quantity One software (Biorad). GST-pull down quantification data were analyzed using GraphPad Prism 4.03 (GraphPad Software). Quantifications presented are means of at least three independent experiments.

### GST-pull Down Competition Experiments

GASP peptide or its corresponding scrambled version was incubated with GST-fused proteins and [^35^S]-labelled GASPs and the binding was analyzed as described for the GST-pull down assay. In a first step, dose-effects of GASP peptides were assessed in competition experiments between [^35^S]-labelled GASP-2 and GASP peptides (concentrations ranging from 1 to 250 µM) for the interaction with GST-ADRB1. Subsequent competition experiments with other GASPs and GPCR C-tails were performed by using a single GASP peptide concentration of 150 µM.

### Production and Purification of Full-length GPCRs

The human ADRB2 and CNR2 receptors were produced using the *P. pastoris* expression system as previously described [15], [16]. After methanol-induced receptor expression, cells were washed with PBS pH 7.0 and resuspended in ice-cold buffer A (50 mM Tris-HCl pH 7.4, 500 mM NaCl, 10% glycerol, 5 mM EDTA, 1 mM PMSF, 2 mM DDT). Cells were then lysed with two cycles of 20 s shaking and 20 s cooling on ice using 0.5 mm glass beads in a FastPrep 24 device. Unbroken cells and cell debris were removed by centrifugation (3000 g, 5 min, 4°C) and the membrane fraction from the supernatant was pelleted by ultracentrifugation (100,000 g, 45 min, 4°C). Membranes were resuspended in buffer B (50 mM Tris-HCl pH 7.4, 500 mM NaCl, 10% glycerol, 1 mM PMSF, 2 mM DTT) using a Dounce homogenizer, and then successively washed with urea (buffer B with 4 M urea) and NaOH (buffer B with 10 mM NaOH), and ultracentrifuged (100,000 g, 45 min, 4°C). Finally membranes were resuspended in buffer B and quantified with the bicinchoninic acid (BCA) method (Pierce).

Approximately 150 mg membrane proteins were extracted by 5 min incubation at room temperature in buffer C (50 mM Tris-HCl pH 7.4, 500 mM NaCl, 10% glycerol, 1 mM PMSF, 2 mM DTT, 50 mM imidazole, 1% (w/v) DDM, 0.1% (w/v) CHS). The solubilized proteins were separated from the remaining membrane fraction by ultracentrifugation (100,000g, 45 min, 4°C) and loaded on a HisTrap 1 ml HP column (GE Healthcare). The column was washed successively with buffer D (50 mM Tris-HCl pH 7.4, 500 mM NaCl, 10% glycerol, 1 mM PMSF, 2 mM DTT, 50 mM imidazole, 0.1% (w/v) DDM, 0.01% (w/v) CHS), buffer D with 2 M NaCl, buffer D with 1 M sodium thyocianate, buffer D with 1% CHAPS, and finally buffer D alone. The proteins were eluted with buffer E (50 mM Tris-HCl pH 7.4, 150 mM NaCl, 300 mM imidazole, 0.1% DDM, 0.01% CHS). Imidazole was removed from the eluted fraction using a HiTrap 5 ml desalting column (GE Healthcare), purified proteins were quantified using the BCA assay (Pierce) and receptor integrity was analyzed by SDS-PAGE.

### Purification of Free GST and GST-fused Central Part of GASP-1

Free GST or GST-fused central part of GASP-1 (AA 380–1073) were purified on an ÄKTApurifier (GE Healthcare) using a GSTrap 4B 1 ml column (GE Healthcare) according to the instructions from the manufacturer. Proteins were eluted with a buffer containing 50 mM Tris-HCl pH 8.0 and 10 mM reduced glutathione. The buffer was finally exchanged for 50 mM Tris-HCl pH 8.0 and glycerol 10% using a HiTrap 5 ml desalting column (GE Healthcare). Purified proteins were quantified using the BCA assay (Pierce), analyzed by SDS-PAGE and stored at −80°C.

### SPR Measurements

A Biacore X100 SPR instrument (GE Healthcare) equilibrated to 25°C and equipped with a Sensor Chip CM5 (GE Healthcare) was used for all SPR measurements.

Affinity purified polyclonal anti-GST antibody (GE Healthcare) was covalently immobilized in two flow cells using Amine Coupling Kit (GE Healthcare) with a running buffer containing 10 mM HEPES pH 7.4, 150 mM NaCl, 0.05% Surfactant P-20 (GE Healthcare) and 3 mM EDTA and a flow rate of 5 µl/min. 50 µg/ml antibody in 10 mM sodium acetate pH 5.0 was injected for 5 min to a surface activated by a 7 min injection of a 1∶1 mixture of 0.4 M 1-ethyl-3-(3-dimetrylaminopropyl) carbodiimide HCl and 0.1 M *N*-hydroxysuccinimide. The remaining active groups were deactivated by a 7 min injection of 1 M ethanolamine HCl pH 8.5. This procedure resulted in immobilization of more than 10000 resonance units (RU ≈ pg/mm^2^ [17]) of anti-GST antibody.

Capture and binding experiments were performed with a running buffer containing 50 mM Tris-HCl pH 7.4, 150 mM NaCl, 0.1% DDM, and 0.01% CHS. Each binding cycle started with capture of GST in the first flow cell by a 0.5–1 min injection of 12.5 nM GST, followed by a 0.5–1 min injection of 94 nM of GST-tagged central domain of GASP-1 in the second flow cell and ended with regeneration by a 2 min injection of 10 mM glycine-HCl pH 2, all at a flow rate of 10 µl/min. Typical capture densities were 50–150 Resonance Units (RU) for GST and 100–200 RU for the central domain of GASP-1. No significant dissociation of the captured proteins was observed in the experimental time frame.

For saturation binding, a range of ADRB2 or CNR2 concentrations were injected after GST and GASP capture. For peptide competition, a fixed concentration of ADRB2 and CNR2 was mixed with a range of GASP peptide or control peptide concentrations and incubated for at least 1 h before injection. Several blank cycles were included for all samples. For the competition curves, an additional blank injection with the appropriate concentration of peptide was injected just before the injection of the peptide and receptor mixture. For all sample injections, the contact time was 3 min, the dissociation phase was 3 min, and the flow rate was 30 µl/min.

The binding curves were double referenced by (i) subtraction of the signal from the GST captured flow cell from the signal of the flow cell with captured central domain of GASP-1 and (ii) subtraction of the appropriate blank injection from the receptor injections with or without peptide [18]. The curves were adjusted for the slightly decreasing GASP capture density during a binding series (20–40 RU from the first to the last GASP capture) by normalization to the response from the first GASP capture. The high peptide concentrations resulted in large spikes at the beginning and end of each injection; these points were clearly artifacts and they were removed for presentation purposes. Endpoint responses were read 20 s after injection end.

The double-referenced dose-response curves were fitted with Biacore X100 Evaluation Software 2.0.1 (GE Healthcare). The dissociation phases were first fitted separately to a one-to-one binding model to obtain the dissociation rate constants. The association and dissociation phases were then fitted simultaneously to a model with two parallel independent one-to-one reactions (heterogeneous ligand), with the dissociation rate constants fixed to the single value found by fitting of the dissociation phases separately. The mass transport contribution was negligible in both data sets. The two association rate constants obtained from these fits were used to estimate the affinity ranges. All curves were fitted by global analysis [18].

### Co-immunoprecipitation Experiments

HEK293 cells stably expressing N-terminal GFP-tagged ADRB1, ADRB2, CALCR and M_1_ or MyrPalm-mYFP were transfected with pcDNA3.1 containing the sequence for a central domain of GASP-1 (AA 380 to 1073) using JetPEI reagent (polyplus transfection) according to the instructions from the manufacturer. After 48 h of expression, cells were washed twice with PBS and lysed in a buffer containing 10 mM Tris-HCl, pH 7.4, 150 mM NaCl, 25 mM KCl, 0.3% Triton X-100 and cOmplete protease inhibitors (Roche) for 1 h at 4°C under agitation. 500 µg of cleared lysates were incubated with 1 µg of a mouse monoclonal anti-GFP antibody (Invitrogen) overnight at 4°C, followed by an incubation with 40 µl of protein A-Sepharose beads for an additional 2 h at 4°C. Beads were then washed five times in lysis buffer and precipitates were resolved on an 8% gel by SDS–PAGE and electrotransferred to immobilon-P membranes (Millipore) in 50 mM Tris-boric acid for 1 h at 300 mA. Membranes were blocked with 5% non-fat powder milk in TBS-Tween (50 mM Tris-HCl pH 8, 150 mM NaCl, 0.4% Tween-20) for 1 h at room temperature. Detection of GFP-tagged receptors was performed by a 2 h incubation of the blots with goat anti-GFP coupled to horseradish peroxidase-conjugated antibody (Abcam; 1∶10,000). For GASP detection, blots were incubated for 1 h with anti-GASP polyclonal serum from rabbit (1∶2,500) and for 1 h with HRP-conjugated anti-rabbit antibody (New England Biolabs; 1∶5,000). For immunodetection of GASP or GFP-tagged receptors directly from cell lysates, 10 µg of proteins were loaded on SDS gels and analyzed following the procedure described above. Finally, membranes were washed in TBS-Tween and detected by chemiluminescence (ECL prime, GE Healthcare).

## Results

### GASPs form a Family of G Protein-coupled Receptor Interacting Proteins

**Figure 1:**
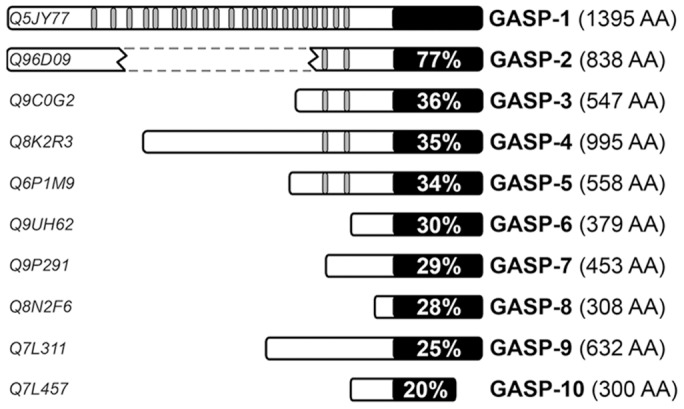
Schematic comparison of GASP family members. Black boxes represent the conserved carboxyl-terminal domain of 250 amino acids. The percentage of identical amino acids shared with GASP-1 is indicated within each box. Small grey boxes represent a highly conserved motif of 15 amino acids that is repeated 22 times in GASP-1 and two times in GASP-2 to -5. The consensus sequence of this motif is: (E/D/G) (E/D) E X (I/L/V/S/T) (I/V/A/F) (G/N) (S/T) W F W (A/V/T/S/D/E) (G/E/R) (E/D/K) (E/D/K/A/Q). For GASP-2, two regions showing significant sequence homology with GASP-1 are separated by a gap represented by dotted lines. GASPs accession numbers from SPtrEMBL database are indicated on the left of the figure.

**Figure 2:**
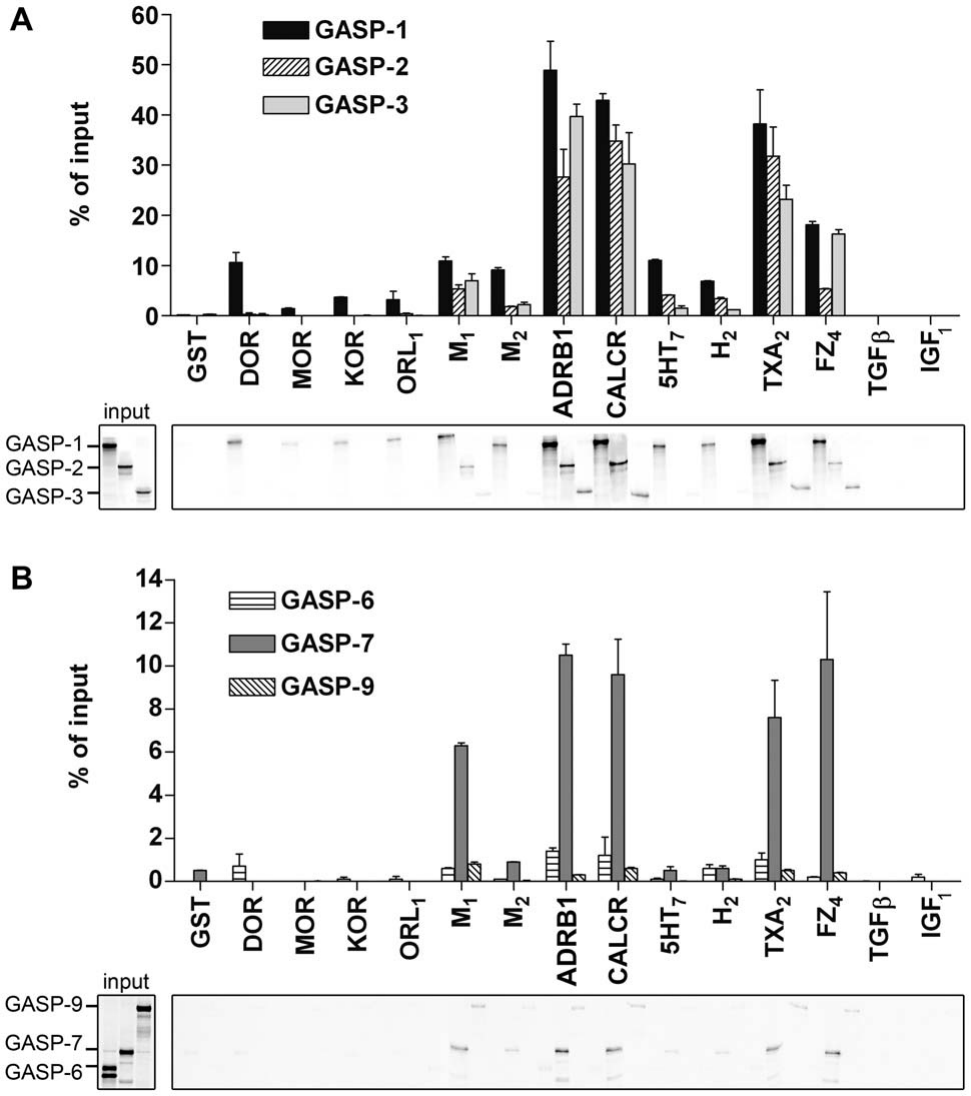
GST-pull down experiments with radiolabelled GASP-1, -2, -3, -6, -7 and -9 and GST-fused receptor C-tails. *A,* GASP-1, -2 and -3 showed medium to strong interactions with some GPCR C-tails but no interaction was detected with the two one-transmembrane receptor C-tails (TGF_β_ and IGF_1_). *B,* GASP-7 showed weak to medium interactions with some GPCR C-tails. GASP-6 and -9 showed very weak interactions with all tested receptors. No interaction was detected with TGF_β_ and IGF C-tails. Data were quantified by Phosphor-imaging. Results are shown as percent of the [^35^S]-GASPs input retained by the GST-fused receptor C-tails and correspond to the mean ± S.E.M of three independent experiments. Lower panels correspond to representative gel images. 5HT_7_, 5-hydroxytryptamine 7 receptor; ADRB1, β_1_ adrenergic receptor; CALCR, calcitonin receptor; DOR, δ-opioid receptor; FZ_4_, frizzled 4 receptor; H_2_, histamine 2 receptor; IGF_1_, insulin growth factor I receptor; KOR, κ-opioid receptor; M_1_, muscarinic M_1_ acetylcholine receptor; M_2_, muscarinic M_2_ acetylcholine receptor; MOR, µ-opioid receptor; ORL_1_, opioid receptor-like 1; TXA_2_, α isoform of the thromboxane A_2_ receptor; TGF_β_, type III transforming growth factor β receptor.

In our previous study we have shown that GASP-1 and GASP-2 interact with the carboxyl-terminal domain of several GPCRs. Based on sequence homology searches, we identified eight additional members of the GASP family ([6], [13]; Figure 1). All members display sequence similarities in their carboxyl-terminal domain (last 250 amino acids) and the first five members contain a repeated motif of 15 amino acids outside this domain, that we named the GASP motif (Figure 1). Moreover, a crosswise comparison of the conserved carboxyl-terminal domain of the different members of the GASP family revealed very high sequence similarities between GASP-1 and GASP-2 as well as high similarities between GASP-6, -7, -8 and -9 (Figure S1). Although GASP-1 and GASP-2 have been shown to interact with GPCRs [6], [7], [10], there is no experimental evidence showing that the other members of the GASP family also interact with these receptors. In order to examine this possibility we performed GST-pull down experiments with radiolabelled GASP-1, -2, -3, -6, -7, and -9 and the cytoplasmic C-tail of twelve GPCRs fused to GST (Figure S2), all comprising the helix 8 that was shown to be critically involved in the interaction with GASP-1. As controls, we used free GST and GST-fused C-tails of the type III transforming growth factor β receptor (TGF_β_) and the insulin growth factor I receptor (IGF_1_), which are non-GPCR receptors. Equivalent amounts of radiolabelled GASPs were incubated with saturating concentrations of GST-fused receptor C-tails immobilized on glutathione-sepharose beads. Radiolabelled proteins that were retained by receptor C-tails were then analyzed by SDS-PAGE and quantified by Phosphor-imaging. As shown in Figure 2, the two non-GPCR receptor C-tails (TGF_β_ and IGF_1_) did not interact with any GASPs (Figure 2A–B). Although sometimes weak, we observed significant interactions of the tested GASPs with several GPCR C-tails.

Based on their interactions with the C-tail of GPCRs, we identified two GASP subfamilies. The first subfamily, including GASP-1, -2, and -3, displayed strong interaction (>10% of GASP input) with most GPCR C-tails tested (Figure 2A). The best GPCR interacting partners were the β_1_ adrenergic receptor (ADRB1), the calcitonin receptor (CALCR), and the α isoform of the thromboxane A_2_ receptor (TXA_2_), which all retained from 30% to 50% of GASP inputs. Furthermore, the atypical frizzled 4 receptor (FZ_4_) also displayed strong interaction with GASP-1 and GASP-3. The other GPCRs displayed medium to low interaction levels (<10% of GASP input). Among the opioid receptors, only DOR showed significant interactions with GASP-1, as shown earlier [6], [7]. The second subfamily, including GASP-6, -7, and -9, showed weak (GASP-7) to very weak (GASP-6 and -9) interactions with GPCR C-tails (Figure 2B). The best interacting partners for GASP-7 were muscarinic M_1_ acetylcholine receptor (M_1_), ADRB1, CALCR, TXA_2_ and FZ_4_ that retained from 6% to 10% of the GASP-7 input. Some receptor C-tails, including DOR, M_1_, ADRB1, CALCR, histamine H_2_ receptor and TXA_2,_ retained around 2% of GASP-6 or GASP-9 inputs, while controls retained less than 1% of both inputs.

Altogether, these results show that in addition to GASP-1 and -2, other members of the GASP family can interact with a wide range of GPCR C-tails. As describe in Figure 1, members of the GASP subfamily 1 contain 22 (GASP-1) or 2 repeated (GASP-2 to -5) GASP motif. Combined with the fact that subfamily 1 displays a higher level of interaction with GPCR C-tails compared to subfamily 2, this observation prompted us to investigate the significance of the GASP motif in the interaction with GPCRs.

### Two Regions of GASP-1 are Involved in the Interaction with the C-tail of GPCRs

In a previous study, we have shown that the carboxyl-terminal region of GASP-1, corresponding to AA 924 to 1395, displays a strong interaction with the DOR C-tail [6]. Within this region, a 250 AA carboxyl-terminal domain displays high sequence similarities with the other GASPs (Figure 1). We therefore hypothesized that this conserved carboxyl-terminal domain could be critical for the interaction of GASPs with GPCRs. To test this hypothesis, we assessed the interaction of three GPCR C-tails, DOR, ADRB1 and M_1_, that display medium to strong interaction with GASPs (Figure 2A), with truncated mutants of GASP-1 in GST-pull down experiments.

**Figure 3:**
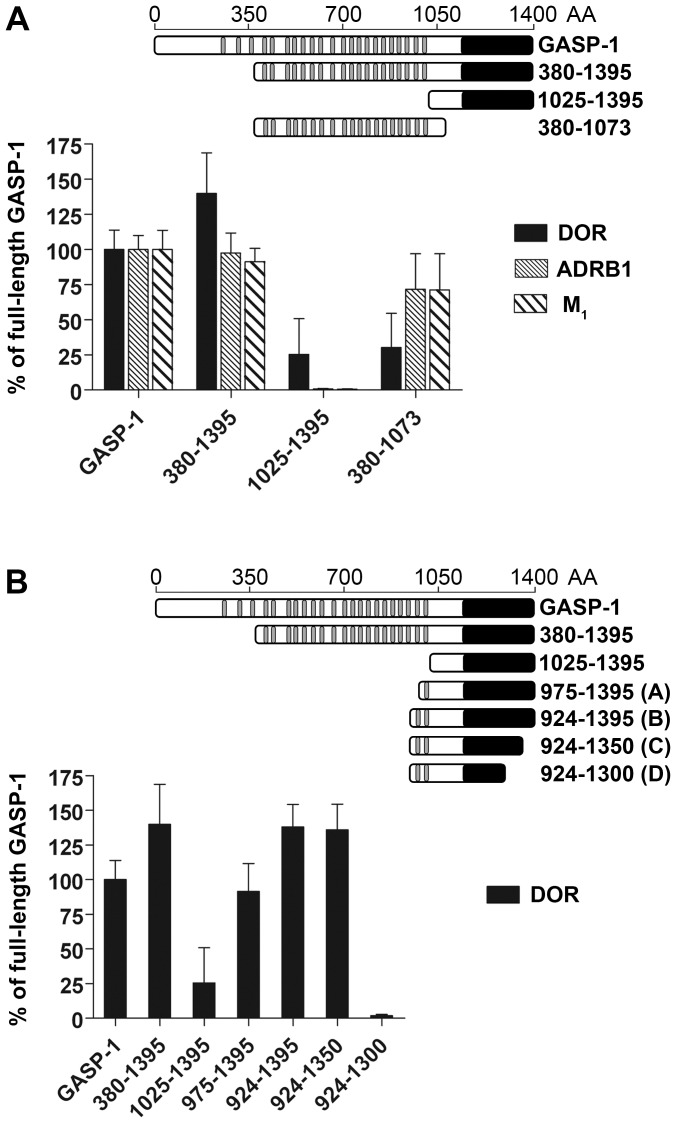
Two portions of GASP-1 are implicated in the interaction with GPCRs. *A,* GST-pull down experiments with three truncated mutants of GASP-1 and DOR, ADRB1 and M_1_ C-tails. Deletion analysis revealed the critical role played by the central part of GASP-1 (380–1073) in the interaction with the DOR, ADRB1 and M_1_ C-tails and especially with ADRB1 and M_1_. Surprisingly, the conserved carboxyl-terminal part of GASP-1 (1025–1395) displayed no significant interaction with these receptor C-tails, except DOR for which a 25% interaction was observed. *B,* GST-pull down experiments with additional truncated mutants of GASP-1 and DOR C-tail. Detailed deletion analysis showed that the interaction with DOR C-tail required the entire carboxyl-terminal part as well as the central part of GASP-1. Results are represented as percent of the full-length GASP-1 interaction and correspond to the mean ± S.E.M of three independent experiments.

In a first set of experiments, we tested three truncated mutants of GASP-1: mutant 380–1395 that lacks the N-terminal part, mutant 1025–1395 corresponding to the conserved C-terminal domain, and mutant 380–1073 corresponding to a central portion of GASP-1 that contains 19 GASP motif. As shown in Figure 3A, DOR, ADRB1 and M_1_ C-tails interacted similarly with full-length GASP-1 and mutant 380–1395, indicating that the N-terminal portion of GASP-1 is not implicated in the interaction with GPCRs. Unexpectedly, the conserved C-terminal domain did not display detectable interactions with the ADRB1 or M_1_ C-tails and interacted only weakly with DOR. Conversely, the central domain of GASP-1 retained around 70% of interaction with ADRB1 and M_1_ C-tails and 30% with DOR. These data suggest that the central domain of GASP-1 is necessary and sufficient for the interaction with ADRB1 and M_1_ receptors, while both the central and C-terminal domains of GASP-1 are important for the interaction with DOR.

In order to delineate more precisely which regions of GASP-1 are important for the interaction with DOR, we evaluated the interaction of the DOR C-tail with four additional truncated mutants of GASP-1 (Figure 3B). The first two were derived from mutant 1025–1395 by extension with 50 or 100 AA at the N-terminus to include one or two GASP motifs (mutants A and B) and the other two were derived from mutant 924–1395, which contains 2 GASP motifs, by deletion of 45 or 95 AA from the C-terminus (mutants C and D). When incubated with DOR C-tail, mutants A and B displayed increasing interaction compared to the C-terminal domain of GASP-1, which were similar to those obtained with full-length GASP-1. Mutant C displayed strong interaction as well, while it was completely lost with mutant D. Altogether, these results indicate that the GASP motifs from the central domain are important to warrant full interaction with DOR and that the integrity of the GASP-1 carboxyl-terminal portion is required for this interaction. From these results we identified two types of interactions between GPCRs and GASP-1: some GPCRs interact exclusively with the central part of GASP-1 (e.g. ADRB1 and M_1_), while others interact with the central and the carboxyl-terminal portions of GASP-1, like DOR.

### Purified Full-length Receptors Bind dose-dependently to the Central Domain of GASP-1

**Figure 4:**
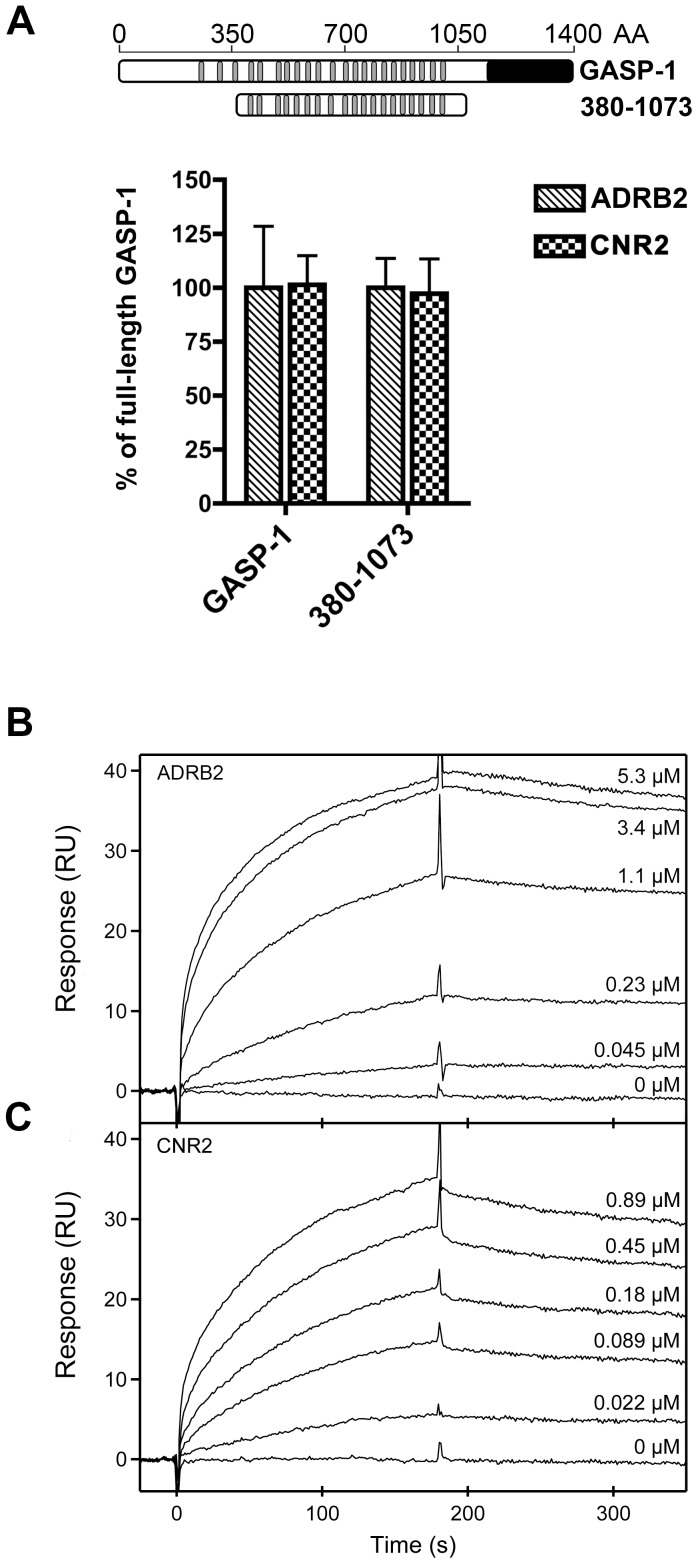
Purified full-length GPCRs dose-dependently bind to the central domain of GASP-1 in SPR experiments. *A*, Interaction of the central domain of GASP-1 compared to the full-length protein with GST-fused ADRB2 and CNR2 C-tails by GST pull down experiments. The results show that both receptors interact *in vitro* with GASP-1 and that the central part of GASP-1 is strongly involved in the interaction with ADRB2 and CNR2. *B*, Binding of a range of concentrations of ADRB2 to the central domain of GASP-1. *C*, Binding of a range of concentrations of CNR2 to the central domain of GASP-1. Overall, we observed a dose-dependent binding of ADRB2 and CNR2 with the central domain of GASP-1. The receptor concentrations are indicated on the figures. All curves are double referenced and corrected for changes in capture density of the central domain of GASP-1. ADRB2, β_2_ adrenergic receptor; CNR2, cannabinoid receptor type 2.

In order to further characterize the importance of the central part of GASP-1 for the interaction with GPCRs, we evaluated the interactions between a central domain of GASP-1 (AA 380 to 1073), which contains 19 GASP motifs, and two purified full-length GPCRs: the β_2_ adrenergic receptor (ADRB2) and the cannabinoid receptor type 2 (CNR2). We first showed that the C-tails of these two receptors interact with GASP-1 in GST Pull-down experiments and that the central domain of GASP-1 is mandatory and sufficient for this interaction (Figure 4A), which is in agreement with previous observation for ADRB1 and M1 (Figure 3A).

Both ADRB2 and CNR2 full-length receptors were produced in *P. pastoris* and purified as previously described [15], [16], while GST-tagged central domain of GASP-1 was purified from BL21 *E. coli* strain (Figure S3). Biacore SPR was used to monitor the binding of GPCRs to GASP-1 in real-time and thus to determine the kinetics and the affinity of the GASP-1–GPCR interactions. The GST-tagged central domain of GASP-1 was first captured with anti-GST antibodies immobilized on a CM5 sensor chip. As a control, free GST was captured in another flow cell of the sensor chip. A range of concentrations of the purified GPCRs was then injected in both flow cells and the interaction was monitored as a function of time. All curves were double referenced by subtraction of the signal from the GST and from the buffer of receptor injections. Using this set-up we observed dose-dependent binding of ADRB2 and CNR2 to the central domain of GASP-1 (Figure 4B–C). The kinetics of the two interactions were very similar: both interactions were very stable and had a slow association rate. For both sets of binding curves, we found complex kinetics in the association phase and a unimodal dissociation phase (Figure S4). Analysis of the dissociation phases showed a dissociation rate constant of *k*_d_ = 4.5±0.1×10^−4^ s^−1^ for ADRB2 and *k*_d_ = 6.5±0.1×10^−4^ s^−1^ for CNR2. The complex association phases did not allow accurate determination of the association rate constants, but we estimated the dissociation constant *K*_d_ to be in the ranges ∼ 14–140 nM for ADRB2 and ∼ 5–50 nM for CNR2.

### The Central Domain of GASP-1 Interacts with GPCRs in Cells

**Figure 5:**
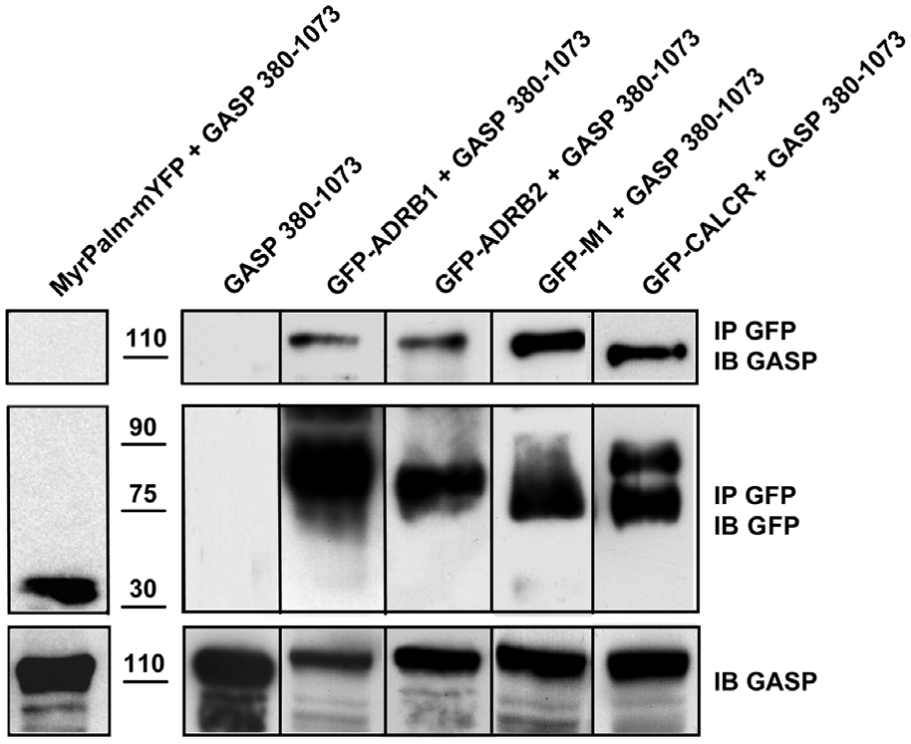
The central domain of GASP-1 co-immunoprecipitates with GPCRs in cells. The central domain of GASP-1 (amino-acids 380 to 1073 of GASP-1 in pcDNA3.1) was transiently transfected in HEK293 cells stably expressing GFP-tagged ADRB1, ADRB2, CALCR or M_1_ receptor. HEK293 cells stably expressing MyrPalm-mYFP and transiently transfected with the central domain of GASP-1 were used as a negative control. The central domain of GASP-1 co-immunoprecipitated with the four different GPCRs while no co-immunoprecipation was observed in cells expressing the central domain of GASP-1 alone or co-expressing this domain with myristoylated-palmitoylated mYFP (MyrPalm-mYFP).

To confirm the relevance of our previous observations we sought to examine the interaction between different GPCRs and the central domain of GASP-1 in a cellular context. To this purpose we performed co-immunoprecipitation experiments from HEK cells stably expressing GFP-tagged ADRB1, ADRB2, CALCR or M_1_ receptor, transfected with the central domain of GASP-1 (AA 380-1073). As expected, the four GFP-tagged GPCRs where mainly localized at the plasma membrane while the central domain of GASP-1 displayed a cytoplasmic distribution that was similar to that observed for the full length GASP-1 [7]. As shown in Figure 5, the central domain of GASP-1 co-immunoprecipitated with the four different GPCRs but not with myristoylated-palmitoylated mYFP (MyrPalm-mYFP), which is targeted to the plasma membrane and particularly enriched in lipid rafts where GPCRs are also preferentially targeted [19]. These data demonstrated that the central part of GASP-1 displays binding activity for full length GPCRs in a cellular context, thus confirming the relevance of our *in vitro* measurements with carboxyl-terminal tails or purified receptors.

### The GASP Motif is Critical for the Interaction of GASPs with GPCRs

**Figure 6:**
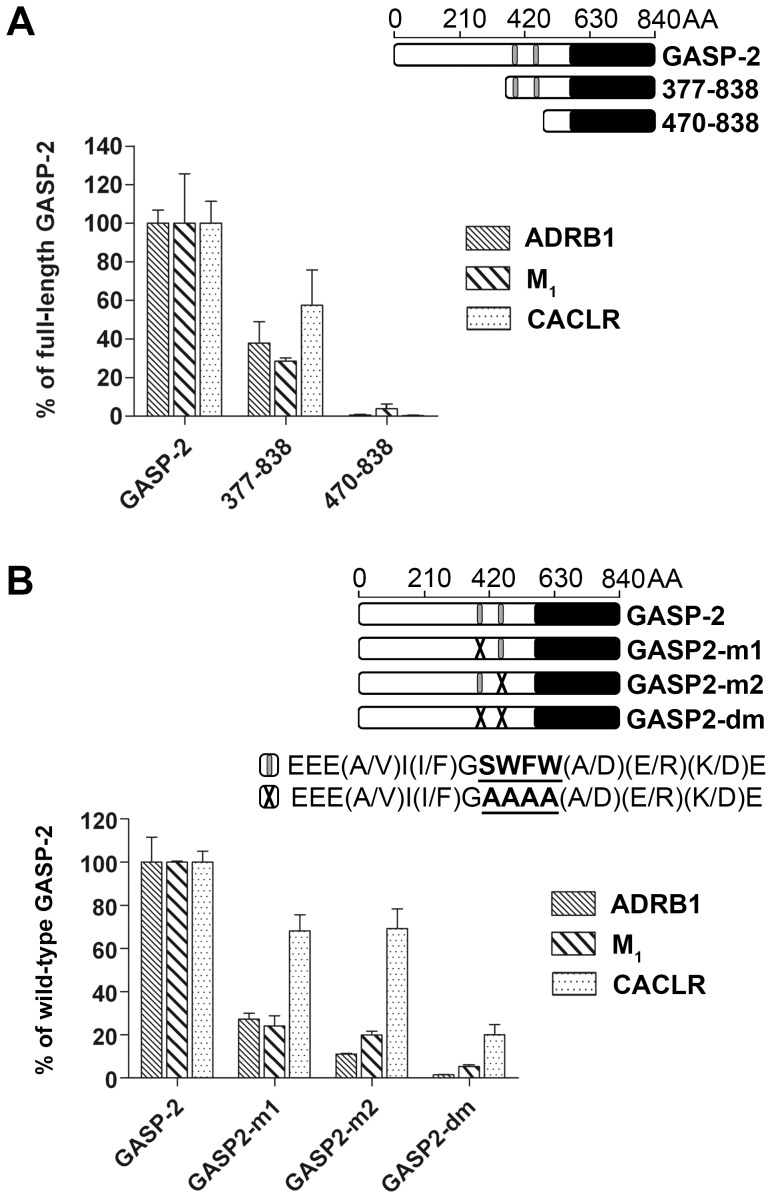
The GASP motif is critical for the interaction of GASP-2 with GPCRs. *A,* GST-pull down experiments with two truncated mutants of GASP-2 and ADRB1, M_1_ and CALCR C-tails. Grey boxes represent the 15 AA GASP motifs. Deletion analysis revealed that the central domain of GASP-2, which contains the two GASP motifs, is critical for the interaction between GASP-2 and ADRB1, M_1_ and CALCR C-tails. *B,* GST-pull down experiments with full-length GASP-2 where one (GASP2-m1 and GASP2-m2) or both GASP motifs (GASP2-dm) were mutated. Grey boxes represent the wild-type motifs and X represent the mutant motifs. Consensus sequences are given for wild-type and mutant motifs. Mutated amino acids are underlined. Site directed mutagenesis analysis of these two repeated motifs showed that they played a crucial role in the interaction of GASP-2 with the three receptor C-tails tested here. Results are shown as percent of the wild-type GASP-2 interaction and correspond to the mean ± S.E.M of three independent experiments.

Results obtained with GASP-1 truncated mutants, SPR and co-immunoprecipation experiments pointed to the GASP motifs from the central domain of GASP-1 as critical elements for interaction with GPCRs. This conserved motif is also present twice in the corresponding central parts of GASP-2 to GASP-5, thus suggesting that it could also play an important role in the interaction between GPCRs and these GASP subfamily members. Thus, we evaluated the interaction of truncated GASP-2 mutants with GST-fused ADRB1, M_1_, and CALCR C-tails, for which we had previously shown a strong interaction with full-length GASP-2 (see Figure 2). As shown in Figure 6A, mutant 377–838, resulting from deletion of the N-terminal part of GASP-2 (upstream from the first repeated motif), displayed interaction with all three receptor C-tails ranging from 30% to 60% compared to the full-length GASP-2. Conversely, mutant 470–838, corresponding to the C-terminal domain of GASP-2, displayed almost no interaction with the three GPCR C-tails tested (Figure 6A). As it was observed for GASP-1, these results suggested that the central portion and the two GASP motifs of GASP-2 are important for its interaction with GPCRs.

In a second step, we focused on the two GASP motifs of GASP-2. As shown in Figure 6B, we replaced the four most conserved residues from these motifs (SWFW) by alanines, either individually (GASP2-m1 and GASP2-m2) or in combination (GASP2-dm). The resulting mutants were probed for their interaction with ADRB1, M_1_, and CALCR C-tails: compared to wild-type GASP-2, both GASP2-m1 and -m2 displayed a strong decrease in their interaction with ADRB1 and M_1_ C-tails, but they retained around 70% interaction with CALCR. For the double mutant, GASP2-dm, almost no interaction was detected with the ADRB1 and M_1_ C-tails and a weak interaction was measured with the CALCR C-tail. Altogether, these results indicated that the GASP motif plays a crucial role in the interaction between GASP-2 and GPCRs. Interestingly, site directed mutagenesis experiments pointed to the highly conserved SWFW sequence within the GASP motif as a key element for interaction with GPCR C-tails.

### A Small Synthetic Peptide Derived from the GASP Motif is Capable of Disrupting Interactions between GASPs and GPCRs

**Figure 7:**
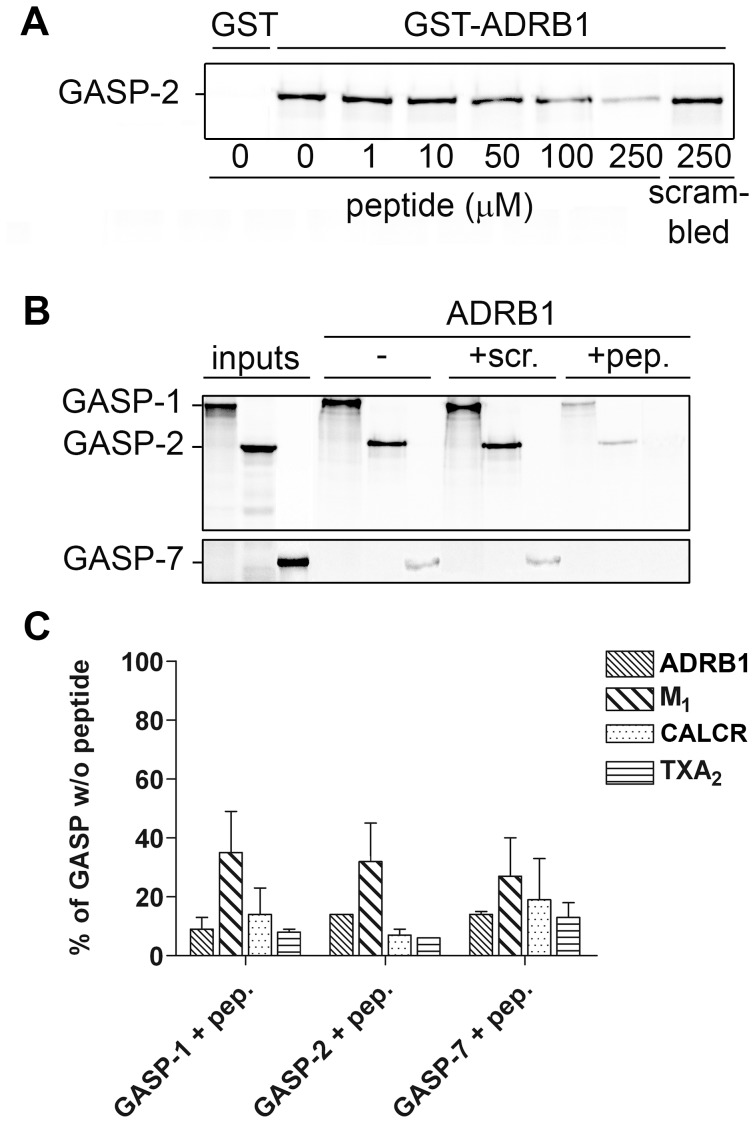
A small synthetic peptide derived from the GASP motif of GASP-2 blocks the interaction between GASPs and GPCR C-tails in GST-pull down experiments. A, GASP peptide competes for the interaction between GASP-2 and GST-fused ADRB1 C-tail. The scrambled peptide displayed no significant effect on the interaction between GASP-2 and ADRB1. *B,* A fixed concentration of GASP peptide (150 µM) inhibits the interaction between GASP-1, -2 or -7 with ADRB1 C-tail, but not the scrambled peptide. *C,* Phosphor-imaging quantification of the competition experiments for the interaction between GASP-1, -2 and -7 and four different receptor C-tails with GASP peptide. A fixed concentration of GASP peptide (150 µM) strongly inhibited interactions of GASPs with ADRB1, M_1_, CALCR and TXA_2_ C-tails. Results are represented as percent of the interaction between the corresponding GASPs and GPCRs in absence of peptide (mean ± S.E.M of three independent experiments).

We further investigated whether small synthetic peptides containing the GASP motif could compete with GASPs for interaction with GPCRs. In a first step, we performed competition experiments with a synthetic peptide of 24 amino acids containing the first GASP motif of GASP-2 (GASP peptide). Peptide concentrations ranging from 1 to 250 µM were tested for their capacity to disrupt the interaction between ADRB1 C-tail and GASP-2. As shown in Figure 7A, increasing amounts of peptide led to a decrease in the amount of GASP-2 that was retained by the ADRB1 C-tail, while addition of the highest dose of a scrambled peptide did not affect this interaction. Inhibitions of 65±6% and 76±6% were obtained with 100 µM and 250 µM GASP peptide, respectively.

In a second step, we used a single peptide concentration (150 µM) in competition experiments with four different receptor C-tails, ADRB1, M_1_, CALCR, and TXA_2_, and three different GASPs, GASP-1 and -2 that contain GASP motifs and GASP-7 that does not. Figure 7B displays a representative experiment for ADRB1 C-tail and the three GASPs. Addition of 150 µM GASP peptide almost completely prevented the interaction between GASP-1, -2, -7 and the ADRB1 C-tail, while scrambled peptide did not. Quantifications revealed that in the presence of 150 µM of GASP peptide, the four receptor C-tails retained between 6% and 35% of GASP-1, -2 and -7 compared to control values (Figure 7C). Altogether, these results confirmed that the GASP motif is mandatory for the interaction of GASPs with GPCRs. Moreover, the interaction between GASP-7, which does not exhibit the GASP motif, and GPCRs was also inhibited by the GASP peptide suggesting that the two GASP subfamilies (with or without GASP motif) most likely interact with the same region of the GPCR carboxyl-terminal domain but with a distinct mode of binding.

**Figure 8:**
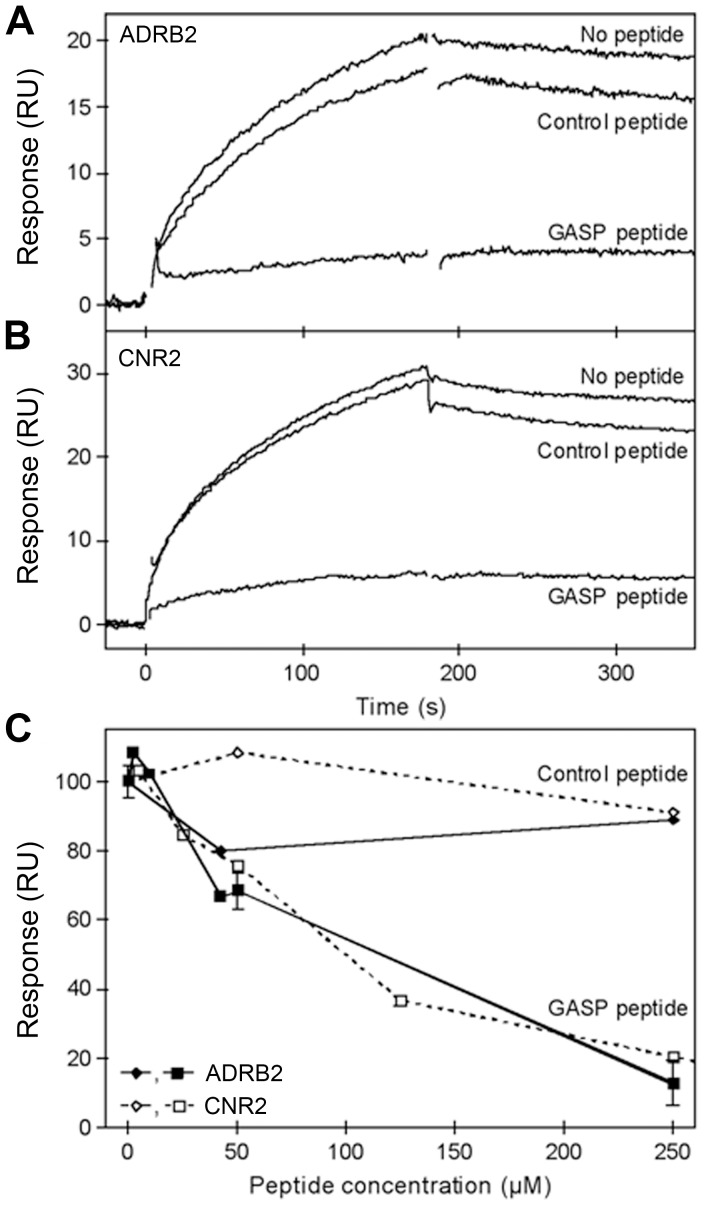
GASP peptide prevents receptor binding to the central domain of GASP-1 in SPR experiments. *A*, Binding of 1.1 µM ADRB2 alone or preincubated with either 250 µM GASP peptide or 250 µM control peptide to captured central domain of GASP-1. *B*, Binding of 0.30 µM CNR2 alone or preincubated with either 250 µM GASP peptide or 250 µM control peptide to captured central domain of GASP-1. All curves are double referenced and corrected for changes in captured GASP density. *C*, Endpoint responses from competition binding curves for 1.1 µM ADRB2 preincubated with a range of concentrations of either GASP peptide (▪) or control peptide (•) and 0.30 µM CNR2 preincubated with either GASP peptide (□) or control peptide (○). The responses are normalized to the endpoint response from an injection with receptor only (0 µM peptide).

Using SPR, we have shown that the central part of GASP-1 can efficiently interact with full-length ADRB2 and CNR2. In a second series of experiments, we examined whether the GASP peptide could also compete with binding of full-length receptors to the central domain of GASP-1. A single concentration of purified ADRB2 or CNR2 were preincubated with different concentrations of peptide before injecting the mixture to a surface with central domain of GASP-1 prepared the same way as for the dose-response experiments (Figure 4). Signals from free GST and from buffer injections were subtracted from the curves. As expected from GST-pull down competition experiments (Figure 7), preincubation with 250 µM GASP peptide strongly decreased the binding of both receptors to the central domain of GASP-1 (Figure 8A–B). This competition was dose-dependent with an IC_50_ estimated to be around 100 µM (Figure 8C). In contrast, a control peptide where the conserved SWFW motif was changed to AAAA only had a minor effect on the interaction (Figure 8). Overall, these results indicate that the interaction between GPCRs and the central part of GASP-1 is specific and that the GASP motifs in the central domain are critical for the interaction with GPCRs.

### Conclusions

In our previous study we have identified a novel family of proteins from which the two first members, GASP-1 and GASP-2, interact with GPCRs [6]. We provide here the first evidence that other members of the GASP family can also interact with the C-tail of GPCRs. Although some members, including GASP-6 and GASP-9, displayed very weak level of interaction (about 1% to 2% of the GASPs input), it was significantly higher than non-specific interaction that was observed with GST alone or some receptor C-tails fused to GST, such as MOR or TGF_β_. As GASP-1 has been shown to modulate the postendocytic sorting of some GPCRs, our results raise the possibility that other GASPs could be implicated in similar functions, thus adding another level of complexity in agonist-induced intracellular trafficking of GPCRs. A key issue will then be to define how the selectivity of interaction between GASPs and GPCRs is achieved in a cellular context.

Among the receptor C-tails that we have tested, ADRB1 is the one that displays the highest level of interaction *in vitro* with GASP-1 and GASP-2. This result is in contrast with previous results showing a low interaction of GASP-1 with ADRB1 C-tail [10]. This discrepancy is most likely due to the fact that we used full-length GASPs to perform our experiments, while Heydorn and collaborators used a truncated form of GASP-1, corresponding to amino-acids 898 to 1395. When we performed GST-pull down experiments with a similar fragment of GASP-1 (corresponding to amino acids 924 to 1395), only 5% of the input was retained by the ADRB1 C-tail (data not shown), which is in agreement with the results of Heydorn et al. [10]. Therefore, our results suggest that the repertoire of GPCRs that interacts with GASP-1 could prove to be even larger than previously anticipated [10].

While numerous GPCRs have been shown to interact with GASP-1 [6], [7], [10], little is known about the molecular mechanisms underlying the GASP-GPCR interaction. In the present study, we show that a small repeated motif of 15 AA present 22 times in GASP-1 and twice in GASP-2 to -5, is critical for the interaction of GASPs with GPCR. Previous studies have only focused on the conserved carboxyl-terminal region of the GASP family in the interaction with GPCRs [6], [7], [10]. Although our results do not exclude a role of this region (see Figure 3B), they clearly show that this motif, that we named “GASP motif”, is mandatory for the interaction of GASPs from subfamily one with GPCRs and represents a new protein-protein interaction motif. These results, together with the fact that GASP-1 contains 22 GASP motifs, suggest that one GASP-1 molecule could interact with several receptors. Moreover, beside their interaction with GPCRs, recent studies have shown that different members of the GASP family can interact with several non-GPCR proteins, including growth factor receptors and ubiquitin ligases [13]. It is therefore tempting to speculate that, like arrestins or multi-PDZ proteins, GASP-1 could function as an adaptor protein assembling GPCRs and other proteins in order to promote receptor function, signaling or trafficking. Further studies are required to evaluate the functional relevance of these interactions. Concerning GASPs from subfamily two that do not contain GASP motifs, although weaker, we also observed interactions *in vitro* with different GPCRs (Figures 2 and 7). Moreover, the interaction of GASP-7 with these different GPCRs was also blocked by a synthetic peptide containing a GASP motif (Figure 7). These data suggest that the two GASP subfamilies (with or without GASP motif) most likely interact with the same region within carboxyl-terminal domain of GPCRs but with a distinct mode of binding. Although the region within GASPs from subfamily 2 that promote the interaction with GPCRs remains to identify, we propose that the conserved carboxyl-terminal domain of the GASP family is involve in this interaction as it is in GASP-1 and DOR interaction (Figures 3A and 3B).

In this study we have set up an assay to examine the interaction between immobilized GASP protein and detergent solubilized full-length GPCRs using SPR. This approach is well-suited for a quantitative study of the interactions between the GASP family of proteins and any GPCR, because (i) most soluble proteins can be expressed as GST fusion proteins and captured by anti-GST antibodies without disturbing their function, (ii) immobilization of GPCRs, which can easily impair their function [20], is avoided and (iii) a minimum of GPCR handling is required. Optical biosensors have previously been used to study the interaction between recombinant GPCRs and G proteins [21]–[24], but this is one of the first times that interactions between full-length GPCRs and another GPCR interacting protein are studied with such techniques. We have determined the dissociation rate constant for the interaction of the central domain of GASP-1 with full-length ADRB2 and CNR2 and estimated the affinity within an order or magnitude. The dissociation rate constants and the affinities of the two receptors are very close, indicating that the mode of interaction with the central domain of GASP-1 is similar. The slow dissociation constants (k_d_ <10^−3^ s^−1^) suggest a high stability of the GASP–GPCR complex. The relatively slow association rate constant found in both cases does however not necessarily mean that the association rate is slow under native conditions (i.e. in cells), since the association rate–in contrast to the dissociation rate–depends on the local concentration of the interacting proteins. In agreement with GST-pull down experiments, SPR competition experiments revealed that the GASP motif and the amino-acids SWFW within this motif are strongly involved in formation of GASP-GPCRs complexes. As GASP-1, arrestins can form stable complexes with GPCRs [25], [26], and have been suggested to play a role in the postendocytic sorting of receptors [7], [27]. Interestingly, cell studies have indicated that arrestins can also form transient complexes with GPCRs and that these receptors are rapidly recycled instead of being degraded or slowly recycled [26]. Whether GASPs can form transient complexes with some GPCRs remains to be shown.

In summary, we have shown here that the GASP family is divided into two sub-families based on their interaction with C-tail of GPCRs: subfamily 1 comprises GASP-1 to -5 that strongly interact with receptor C-tails and contain a small repeated motif, the GASP motif, while sub-family 2 includes GASP-6 to -10 that weakly interact with the receptor C-tails and does not contain the GASP motif. We also report here the first molecular characterization of the interaction between GASPs and GPCRs. Our data cleary demonstrate that the GASP motif mediates the interaction of GASPs with G protein-coupled receptors and that a small peptide containing this motif is capable of preventing the interaction of GASPs with receptor C-tails and also full-length GPCRs. This study clearly highlight that we have identified a novel protein-protein interacting motif that is implicated in GPCR interactions and might be a new target for investigation of the role played by GASP in the modulation of the activity of GPCRs in vivo.

## Supporting Information

Figure S1 **Crosswise comparison of the conserved carboxyl-terminal domain of GASPs.** Red color corresponds to sequence identity between 90% and 100%, orange dark between 75% and 90%, orange light between 45% and 75%, blue dark between 25% and 44% and blue light less than 25%. In addition to figure 1, this table shows that all GASPs display sequence similarities in their carboxyl-terminal tail and reveals very high sequence similarities between GASP-1 and GASP-2 as well as high similarities between GASP-6, -7, -8 and -9.
(DOC)

Figure S2 **GST-fusions of GPCR C-tails used in GST-Pull down experiments.** Purified proteins were separated by SDS-PAGE and stained with coomassie blue.
(DOC)

Figure S3 **Purification of the central domain of GASP-1, ADRB2 and CNR2.** Purified proteins were separated by SDS-PAGE and stained with coomassie blue. *A*. line 1: crude extract, line 2: cleared lysate, line 3: purified central domain of GASP-1. *B.* line 4: membrane proteins of *P. pastoris* expressing ADRB2, line 5: solubilized membrane proteins, line 6: purified ADRB2. *C.* line 7: membrane proteins of *P. pastoris* expressing CNR2, line 8: solubilized membrane proteins, line 9: purified CNR2. Arrowheads indicated purified proteins.
(DOC)

Figure S4 **Overlay of GASP–GPCR saturation binding curves with fit curves.** Binding of the central domain of GASP-1 to ADRB2 and CNR2 monitored with SPR. *A*, *C*. Overlay of the dissociation phase of the central domain of GASP-1 binding to ADRB2 (A) and CNR2 (C) with fit curves. *B*, *D*. Overlay of the full binding curves for the central domain of GASP-1 binding to ADRB2 (B) and CNR2 (D) with fit curves. In addition to the dose-dependant binding of ADRB2 and CNR2 to the central domain of GASP-1, the dissociation phase revealed a stable interaction between the central domain of GASP-1 and the GPCRs.
(DOC)
