# Supplementary material for: Identification of a Novel Protein-Protein Interaction Motif Mediating Interaction of GPCR-Associated Sorting Proteins with G Protein-Coupled Receptors
Source: PLoS One. 2013 Feb 18;8(2):e56336. doi: 10.1371/journal.pone.0056336 (PMC3575409; doi:10.1371/journal.pone.0056336)

**Supplemental figure S1. Crosswise comparison of the conserved carboxyl-terminal domain of GASPs.** Red color corresponds to sequence identity between 90% and 100%, orange dark between 75% and 90%, orange light between 45% and 75%, blue dark between 25% and 44% and blue light less than 25%. In addition to figure 1, this table shows that all GASPs display sequence similarities in their carboxyl-terminal tail and reveals very high sequence similarities between GASP-1 and GASP-2 as well as high similarities between GASP-6, -7, -8 and -9.


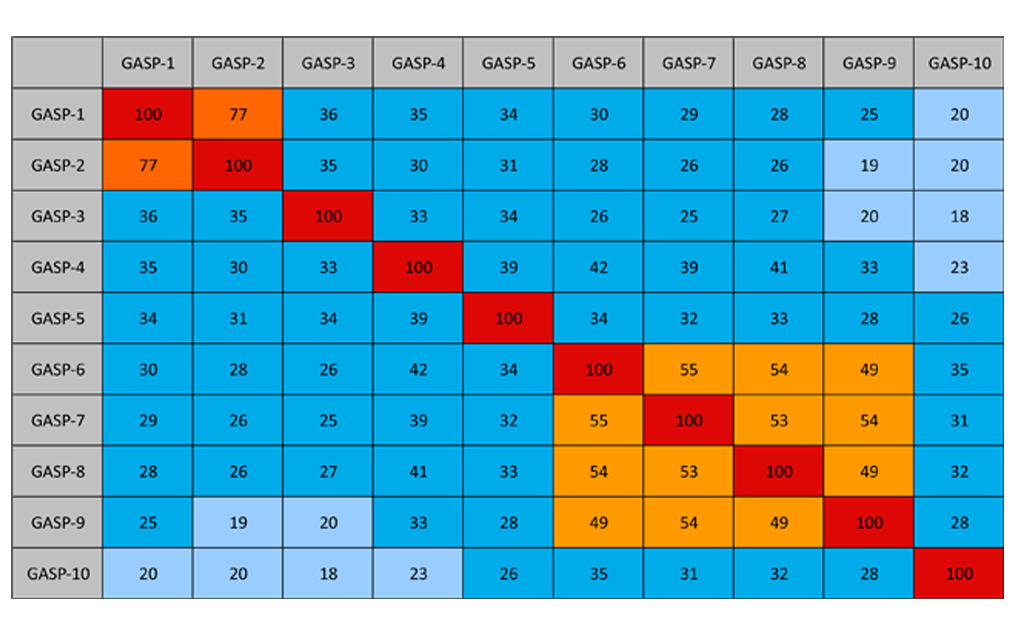

Supplement: Figure S1 — Crosswise comparison of the conserved carboxyl-terminal domain of GASPs. Red color corresponds to sequence identity between 90% and 100%, orange dark between 75% and 90%, orange light between 45% and 75%, blue dark between 25% and 44% and blue light less than 25%. In addition to figure 1, this table shows that all GASPs display sequence similarities in their carboxyl-terminal tail and reveals very high sequence similarities between GASP-1 and GASP-2 as well as high similarities between GASP-6, -7, -8 and -9. (DOC) [file pone.0056336.s001.doc]
