# Supplementary material for: Identification of a Novel Protein-Protein Interaction Motif Mediating Interaction of GPCR-Associated Sorting Proteins with G Protein-Coupled Receptors
Source: PLoS One. 2013 Feb 18;8(2):e56336. doi: 10.1371/journal.pone.0056336 (PMC3575409; doi:10.1371/journal.pone.0056336)

**Supplemental figure S2. GST-fusions of GPCR C-tails used in GST-Pull down experiments.** Purified proteins were separated by SDS-PAGE and stained with coomassie blue.


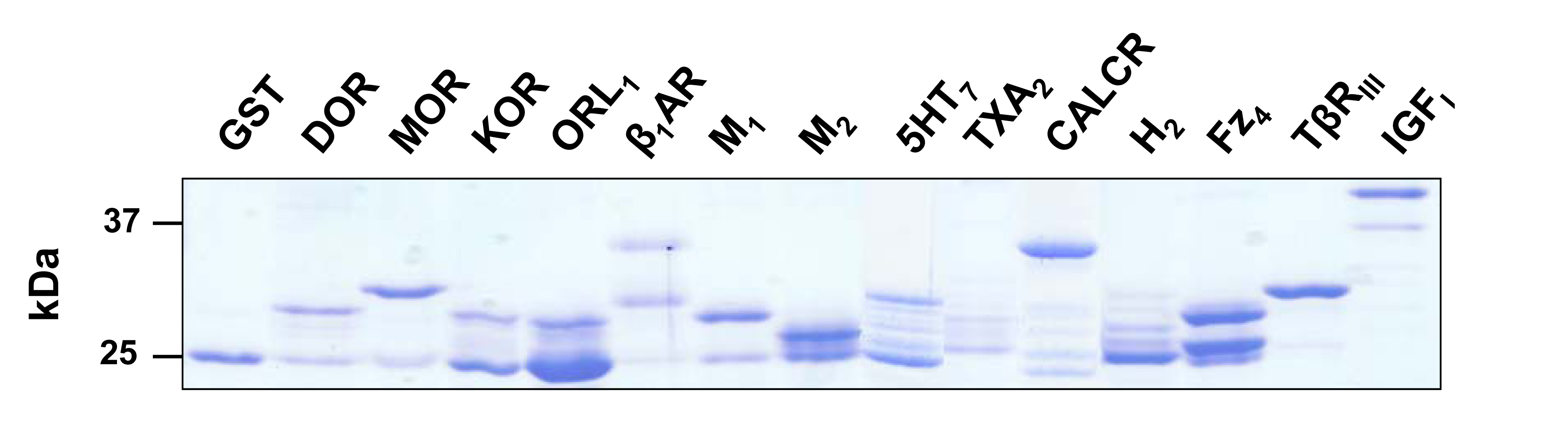

Supplement: Figure S2 — GST-fusions of GPCR C-tails used in GST-Pull down experiments. Purified proteins were separated by SDS-PAGE and stained with coomassie blue. (DOC) [file pone.0056336.s002.doc]
