# Supplementary material for: Identification of a Novel Protein-Protein Interaction Motif Mediating Interaction of GPCR-Associated Sorting Proteins with G Protein-Coupled Receptors
Source: PLoS One. 2013 Feb 18;8(2):e56336. doi: 10.1371/journal.pone.0056336 (PMC3575409; doi:10.1371/journal.pone.0056336)

**Supplemental figure S3. Purification of the central domain of GASP-1, ADRB2 and CNR2.** Purified proteins were separated by SDS-PAGE and stained with coomassie blue. *A*. line 1: crude extract, line 2: clarified lysate, line 3: purified central domain of GASP-1. *B.* line 4: membrane proteins of *P. pastoris* expressing ADRB2, line 5: solubilized membrane proteins, line 6: purified ADRB2. *C.* line 7: membrane proteins of *P. pastoris* expressing CNR2, line 8: solubilized membrane proteins, line 9: purified CNR2. Arrowheads indicated purified proteins.


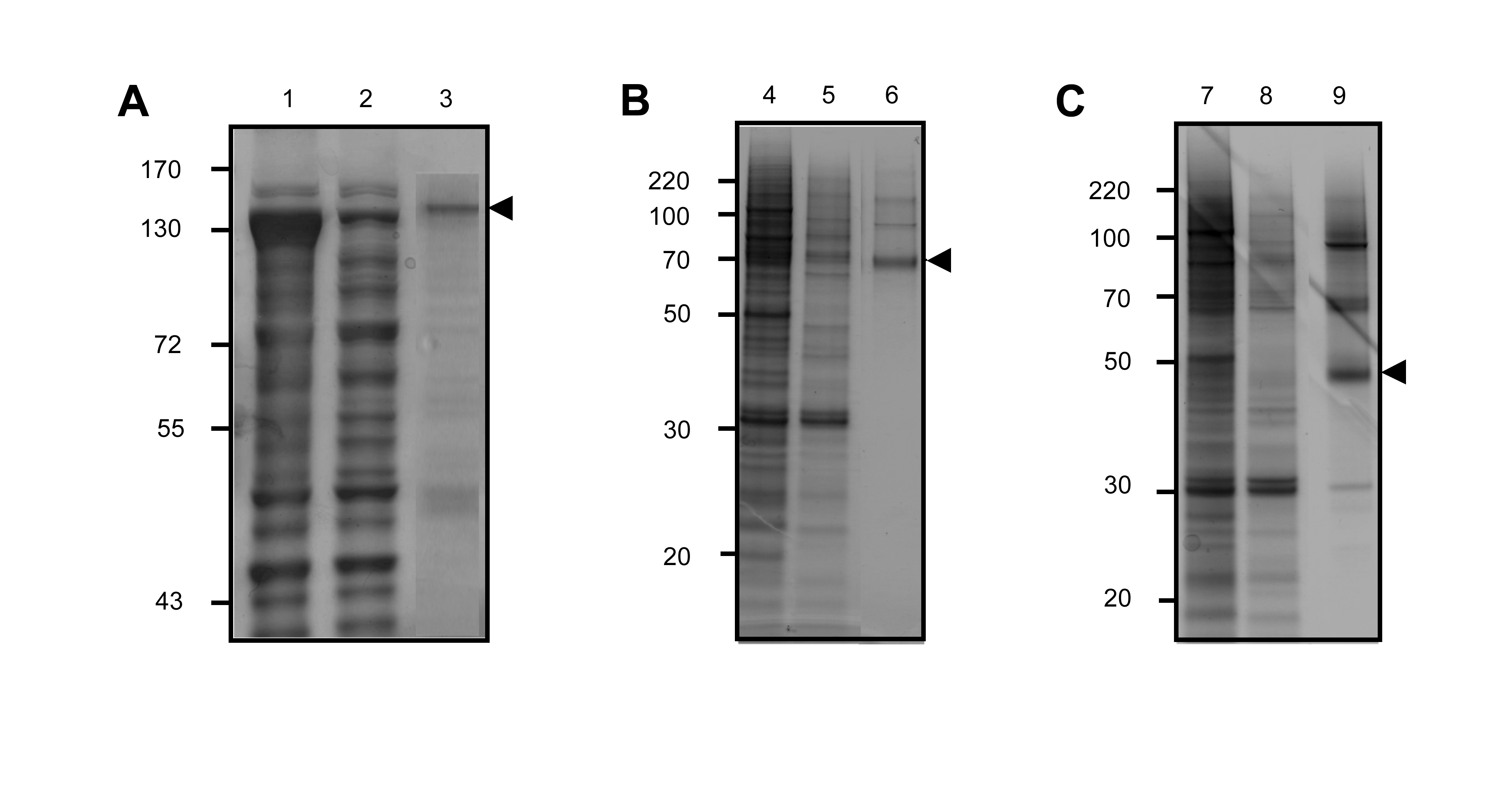

Supplement: Figure S3 — Purification of the central domain of GASP-1, ADRB2 and CNR2. Purified proteins were separated by SDS-PAGE and stained with coomassie blue. A. line 1: crude extract, line 2: cleared lysate, line 3: purified central domain of GASP-1. B. line 4: membrane proteins of P. pastoris expressing ADRB2, line 5: solubilized membrane proteins, line 6: purified ADRB2. C. line 7: membrane proteins of P. pastoris expressing CNR2, line 8: solubilized membrane proteins, line 9: purified CNR2. Arrowheads indicated purified proteins. (DOC) [file pone.0056336.s003.doc]
