# Supplementary material for: Identification of a Novel Protein-Protein Interaction Motif Mediating Interaction of GPCR-Associated Sorting Proteins with G Protein-Coupled Receptors
Source: PLoS One. 2013 Feb 18;8(2):e56336. doi: 10.1371/journal.pone.0056336 (PMC3575409; doi:10.1371/journal.pone.0056336)

**Supplemental figure S4. Overlay of GASP–GPCR saturation binding curves with fit curves.** Binding of the central domain of GASP-1 to ADRB2 and CNR2 monitored with SPR. *A*, *C*. Overlay of the dissociation phase of the central domain of GASP-1 binding to ADRB2 (A) and CNR2 (C) with fit curves. *B*, *D*. Overlay of the full binding curves for the central domain of GASP-1 binding to ADRB2 (B) and CNR2 (D) with fit curves. In addition to the dose-dependant binding of ADRB2 and CNR2 to the central domain of GASP-1, the dissociation phase revealed a stable interaction between the central domain of GASP-1 and the GPCRs.


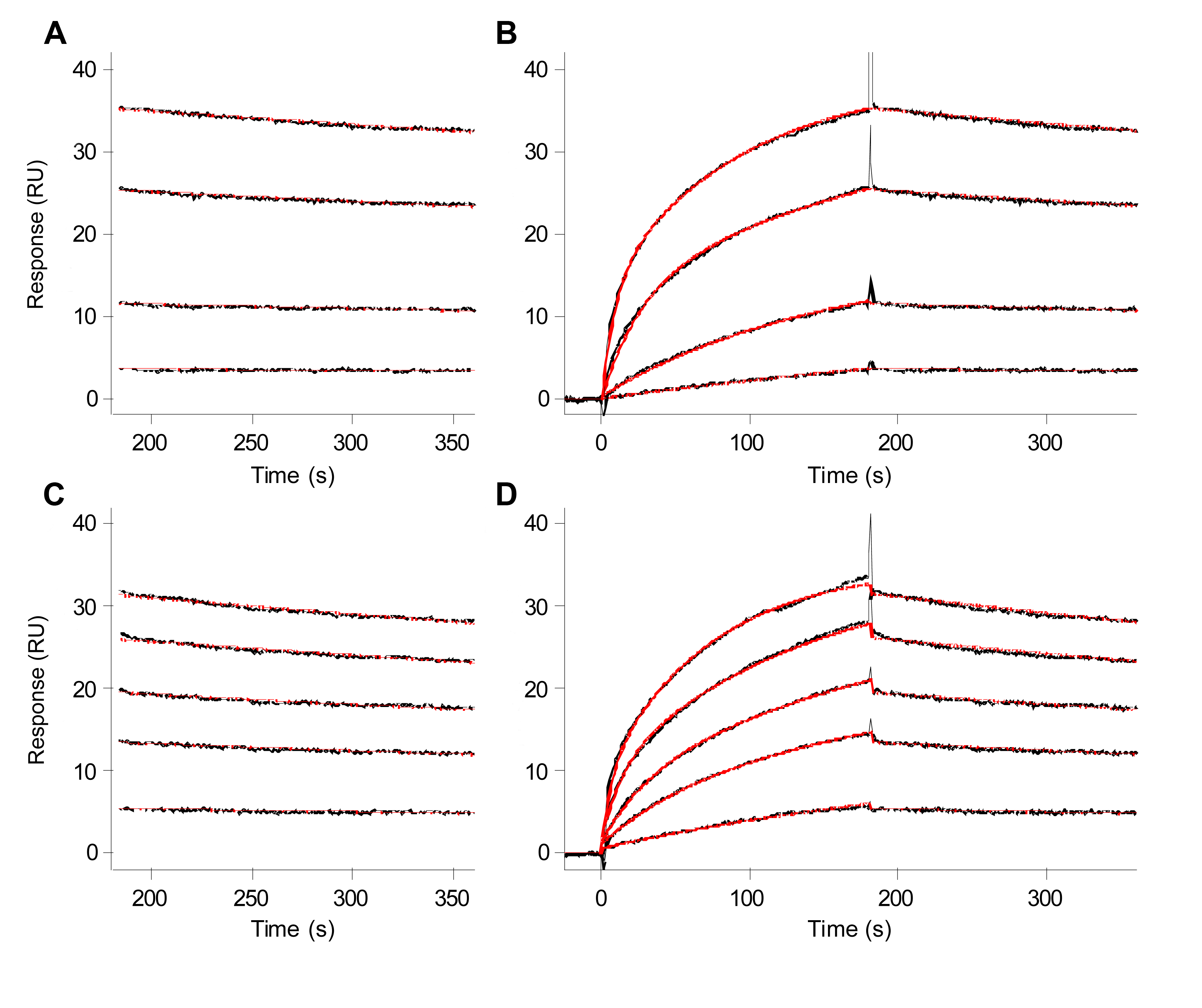

Supplement: Figure S4 — Overlay of GASP–GPCR saturation binding curves with fit curves. Binding of the central domain of GASP-1 to ADRB2 and CNR2 monitored with SPR. A, C. Overlay of the dissociation phase of the central domain of GASP-1 binding to ADRB2 (A) and CNR2 (C) with fit curves. B, D. Overlay of the full binding curves for the central domain of GASP-1 binding to ADRB2 (B) and CNR2 (D) with fit curves. In addition to the dose-dependant binding of ADRB2 and CNR2 to the central domain of GASP-1, the dissociation phase revealed a stable interaction between the central domain of GASP-1 and the GPCRs. (DOC) [file pone.0056336.s004.doc]
